# Models of care for frail older persons who present to the emergency department: a scoping review protocol

**DOI:** 10.1186/s13643-020-01534-z

**Published:** 2020-12-05

**Authors:** Ya-Ling Huang, Megan McGonagle, Rebecca Shaw, Julie Eastham, Nemat Alsaba, Julia Crilly

**Affiliations:** 1 Department of Emergency Medicine, Gold Coast Hospital and Health Service, Gold Coast University Hospital, 1 Hospital Blvd, Southport, QLD 4215 Australia; 2 Bond University, 14 University Dr, Robina, QLD 4226 Australia; 3 School of Nursing and Midwifery, Griffith University, 1 Parklands Dr, Southport, QLD 4215 Australia; 4 Menzies Health Institute Queensland, Griffith University, G40 Griffith Health Centre, Level 8.86 Gold Coast Campus, Griffith University, QLD 4222 Australia

**Keywords:** Frailty, Aged, Emergency service, Hospital, Review

## Abstract

**Background:**

People aged ≥ 65 years comprise around 1 in 5 emergency department (ED) presentations. Many of these presentations occur due to complications associated with chronic diseases and frailty. This review aims to provide a comprehensive understanding of available research regarding models of care for frail older people presenting to the ED.

**Methods:**

The Joanna Briggs Institute scoping review framework will be used to guide this review. Literature searches will be conducted in the following electronic databases (from January 2009 onwards): CINAHL via EBSCOhost, Ovid MEDLINE, Embase, SocINDEX. Grey literature will be identified through searching Google Scholar. This review will consider primary research studies (including observational and interventional studies) published in English on models of care for frail older people (aged ≥ 65) presenting to the ED. Two researchers will independently screen all citations, full-text articles, and abstract data. Potential disagreements will be resolved through discussion with a third researcher. Data extracted from included studies will include the following: author(s), year of publication, country, research design and aim, time frame of the study, study population and sample size, data collection methods, definition of frailty, model of care, and key findings that pertain to the ability to inform this review. The strength of the body of evidence will be assessed using the National Health and Medical Research Council level of evidence hierarchy body of evidence matrix. Data will be presented in a tabular format and accompanied by a narrative that describes the characteristics of the body of literature.

**Discussion:**

Despite the increased number of ED presentations for frail older people, there has been no synthesis of the sources of evidence of model of care for frail older people in the setting of emergency care. The results of this scoping review will provide an overview of different models of care and help inform future research in the development of models of care for frail older persons, tailored to the healthcare system in the emergency context.

**Systematic review registration:**

This scoping review has been registered in the Open Science Framework (osf.io/h2t94).

**Supplementary Information:**

The online version contains supplementary material available at 10.1186/s13643-020-01534-z.

## Background

Worldwide, the population of people aged over 60 is rapidly accelerating from 900 million in 2015 to an estimated 2 billion by 2050 [1]. In 2015, the proportion of people aged 65 years and over (of the total population) was similar in some countries: 15% in Australia, the USA, and New Zealand respectively, 16% in Canada, and 18% in the UK, and higher in other countries: 20% in Greece, 23% in Italy, and 26% in Japan [2]. The most challenging expression of aging is the state of frailty, which develops as a consequence of age-related decline in multiple physiological systems [3]. The prevalence of frailty increases steadily with age from 4 (≥ 65) to 26% (> 85 years) [4].

A recent systematic review has reported noted increases in certain types of emergency department (ED) presentations which include those with urgent and complex needs, low-acuity presentations, and presentations by older people in American, Netherland, Pakistan, Australian, Canadian, and Japanese studies [5]. For example, people aged 65 and over comprised 22% of the eight million ED presentations made in 2017–2018 in Australia [6]. These presentations made by older adults often result from complications associated with certain diseases and frailty [1, 2, 4, 7]. ED presentation can also occur due to shortfalls in access to primary healthcare and management of care in residential aged care facilities (RACFs) [8]. The increase in ED presentations of older people with complex and chronic conditions also worsens the ongoing issue of ED crowding which in turn has a significant impact on patient outcomes and inability of staff to adhere to guideline-recommended treatment [5]. Therefore, appropriate screening for early identification of frailty in patients and care delivery can help to reduce the complications and morbidity associated with frailty.

Several models of care have been outlined in the literature to provide a comprehensive assessment and management of frail older people who present to the ED. These models of care include offering in-home or outreach services, providing prioritization or geriatric-focused care in the ED, and enhancing primary care [9]. More recent models of care for older people in the ED include the Geriatric Emergency Department Intervention (GEDI) [10] and Geriatric Emergency Department Innovations in Care through Workforce, Informatics and Structural Enhancements (GEDI WISE) models [7, 11]. Other models of care that involve interagency engagement between the ED and aged care facilities include Aged Care Services Emergency Program [12], Aged Care Emergency Service [13], and Comprehensive Aged Residents Emergency and Partners in Assessment, Care and Treatment (CARE-PACT) [14]. The differences between models of care can vary based on the underpinning structure of the health service, government initiatives, guidelines, resources, and population. Noted benefits of such models of care designed to improve care for frail older people who present to the ED include reduced ED length of stay, reduced complications associated with ED presentation, and the prevention of inappropriate hospitalizations [8]. To our knowledge, previous review protocols [15–19] and systematic/narrative reviews [20–22] exist on this topic. These reviews primarily focused on (i) outcomes, costs, and implementation factors of ED interventions for older people [15]; (ii) ED-based geriatric case management models [16, 17, 20]; (iii) ED community transition strategies for older people [21]; (iv) frailty measures used in pre-hospital and ED [22]; (v) nurse-led interventions [18]; and (vi) geriatrician-led interventions in the ED [19].

### Aim and review questions

The aim of this scoping review is to provide a comprehensive understanding of available research related to models of care for frail older people who present to the ED as no existing or ongoing scoping review on the topic of the evidence of frailty and models of care for older people in the emergency settings have been identified in the literature. The main research question using the Population-Concept-Context (PCC) framework to guide this scoping review is: What is the research evidence available regarding models of care (concept) for frail older persons (population) in the ED (context)? Sub-questions underpinning this overarching question included the following: (1) How is frailty defined in the ED setting for older people? (2) What are the demographics, clinical profiles, care delivery, and outcomes for frail older patients who present to the ED? (4) What published screening tools exist for frail older people who present to the ED? (5) What published models of care exist targeted for frail older people who present to the ED?

## Methods

The scoping review protocol has been registered within the Open Science Framework database (registration number: osf.io/h2t94) and is being reported in accordance with the reporting guidance provided in the Preferred Reporting Items for Systematic Reviews and Meta-Analyses Protocols (PRISMA-P) statement [23, 24] (see checklist in Additional file 1). The proposed scoping review will be reported in accordance with the reporting guidance provided in the Preferred Reporting Items for Systematic Reviews and Meta-analyses (PRISMA) extension for Scoping Reviews (PRISMA-ScR) [25]. This scoping review will be conducted in accordance with the Joanna Briggs Institute (JBI) scoping review methodology [26]. This includes using the framework of PCC and the three-step search strategy to guide the review process [26]. The definition of terms used is presented in Table 1.

**Table 1:** Definition of terms

Frailty	Frailty is defined as a clinically recognizable state of increased vulnerability as a result of multiple physiologic system deterioration of reserve and functional capacity at older ages such that the ability to cope with daily or acute stressors is comprised [27]. The characteristics of frailty mostly include decline in mobility (i.e., gait speed), physical activity, balance, muscle strength, endurance, motor processing, cognition, and nutrition (i.e., loss of weight) [19, 28].
Older persons	People aged 65 years old and over [29].
Frail older persons	Frail older persons are recognized at greatest risk of adverse outcomes, such as decline in disability, institutionalisation, and death. They are more likely to present with a geriatric syndrome (i.e., delirium and falls) [28]. They also require care from different levels (i.e., gerontology, geriatrics, rehabilitation, internal medicine, nursing, and social work) and integrated and coordinated care [19].
Emergency department	It is a physical location which receives, triages, stabilizes, and provides acute care to patients who require resuscitation, emergent, urgent, semi-urgent, or less-urgent conditions [30].
Model of care	A model of care is designed to provide faster access to safe and quality emergency care. This assists hospitals to meet the National Emergency Access Targets (NEAT) and to improve patient experience [31]. Patient model of care in the emergency department include but are not limited to: triage system (i.e., medical-led triage and nursing assessment team), resuscitation (including trauma), early emergency department senior assessment and streaming, fast track and rapid assessment team, dedicated assessment areas, short stay/observation units, medical assessment units [30–32].

### Eligibility criteria

The PCC framework will be used to align the study selection with the research question [26]. This review will consider all observational and interventional studies that include the population of frail older people presenting to the ED. Frail older people will be referred to as either: “frail,” “frail elderly,” “frail older adults,” “geriatric,” or “aged” and people aged ≥ 65 years. The concept is model of care. Model of care is referred to as delivery of health care provided within the emergency health services to manage and improve care of the frail older people such as medical-led or nursing-led triage/assessment team, fast track and rapid assessment team, and dedicated assessment areas for frail older people. The context of this study setting is the ED. ED includes any facility specializing in emergency medicine.

For this scoping review, we will include original research that investigates model(s) of care for frail older people presenting to the ED. This review will consider published peer-review primary research articles to answer the review question. Studies published in the English language after January 2009 will be included in this review as this review aims to capture the studies published over the last 10 years to reflect the contemporary review of articles. Studies will be excluded based on the following criteria: not published in English, published before 2009, invalid study type (not primary research, i.e., methodology paper/research protocol, review paper, case report, discussion paper, studies with no abstract), thesis, editorial, conference abstract, and duplicates. Also excluded will be studies that are not relevant to frailty and/or only focus on specific types of disease (as the scope of this review focuses on the whole spectrum of frailty care for older people), the study population is not older people aged ≥ 65, and study setting is not in the ED.

### Information source and search strategy

A three-step search strategy will be used in this review [26]. The electronic databases to be searched for published literature (from January 2009 onwards) will include CINAHL via EBSCOhost, Ovid MEDLINE, Embase, and SocINDEX. Grey literature will be identified through searching Google Scholar. The search strategies with search terms for included databases will be developed and performed in consultation with an information scientist (a research and teaching librarian).

In step one, the initial limited search will include a search of Ovid MEDLINE and CINAHL via EBSCOhost databases with keywords (i.e., accident and emergency, A&E, geriatric, elderly, or model of care) and subject headings (i.e., health care delivery, frail elderly, aged, or emergency medical services). This initial search will be followed by an analysis of the text words contained in the title, abstract, and subject headings of retrieved articles relevant to the topic. In step two, a second search using the refined search terms which are tailored to databases of CINAHL via EBSCOhost, Ovid MEDLINE, Embase, and SocINDEX will be undertaken in all included databases and Google Scholar. A draft search for CINAHL via EBSCOhost and Ovid MEDLINE is presented in Additional file 2. In step three, the reference list of identified articles will be searched for additional studies.

### Selection of sources of evidence

After the search is completed, all citations will be imported to EndNote X9 [33] and duplicates will be removed. Two independent researchers (YLH, MM) will screen titles and abstracts for inclusion criteria specified in this protocol. A third researcher (RS) will moderate where agreement is not initially achieved. The studies identified through the title and abstract review will be uploaded in full to EndNote library. These studies will then be reviewed by two reviewers (YLH, MM) to determine their inclusion based on the study inclusion criteria. Any disagreements on full-text inclusion arising during screening between two independent reviewers (YLH, MM) will be moderated by a third researcher (RS). Details for the reasons for exclusion will be noted in the final report. A PRISMA flow diagram showing details of studies included and excluded at each stage of the study selection process will be provided [23].

### Data extraction

The Microsoft Word software will be used for data extraction [34]. Data charting forms will be created in the Microsoft Word and be piloted initially on a small number of included articles by one researcher (YLH). Data will be independently extracted by one researcher (YLH) and crossed check against original articles by a second researcher (JE) to ensure the validity of the extracted data. Potential conflicts will be resolved via discussion, and a third researcher (JC) may be included to moderate the process for determining results if required. Authors of primary publications will be contacted for clarification of reported data if required. Data extracted from included studies will include the information that aligns with the research questions. This includes (i) study characteristics (authors, year, country, research design and aim, time frame of the study, study population and sample size, data collection methods), (ii) definition of frailty, (iii) demographics (age, gender, place of residence), clinical profiles (model of arrival, reasons for presentation, time of day, triage category, ICD-10-CM diagnosis code), care delivery (referrals, consultations, follow-up, diagnostic tests), and outcomes (waiting time to be seen by a doctor, ED length of stay, discharged/admitted, mortality), (iv) screening tools for assessment of frail older people within the model of care, (v) types of model of care for frail older people presented to the ED. A draft of the data collection form is provided in Additional file 3.

### Critical assessment for level of evidence

While it is not compulsory, it is encouraged to perform critical assessment for studies in a scoping review. As there is no JBI assessment tools available for scoping reviews, the National Health and Medical Research Council (NHMRC) Level of Evidence Hierarchy Body of Evidence Matrix [35] will be used to present the strength of the body of evidence. Confidence in cumulative evidence will be assessed by two independent researchers (YLH, NA). If there is any disagreement, a moderation process will be conducted with three researchers (YLH, NA, JC).

### Data synthesis

The Microsoft Word software [34] will be used for data management and presentation. The scoping review results will be synthesized into a narrative summary which aligns with study aim, review questions, and eligibility criteria (PCC framework) and will be thematically sorted based on these criteria. Quantitative data of included articles will be summarized as numerical counts. The extracted data will be presented in tabular form which will be developed and refined throughout the data extraction. A narrative summary will accompany the tabulated results and describe how the results relate to the model(s) of care for frail older people presenting to the ED. Suggestions for future research based on the study findings will also be summarized.

## Discussion

The main goal of this scoping review is to map the existing research evidence with regards to models of care for frail older people presenting to the ED and provide a narrative summary of the extracted data reflected in our review question. With the increase in ED presentations of older people with complex needs, several models of care for frail older people presenting to the ED (i.e., GEDI, GEDI WISE, CARE-PACT) have been reported; however, these have varying features due to different underlying factors (i.e., population, health services, policy, guidelines, and resources). The results of this review will firstly help describe the definition of frailty in the setting of emergency care. Secondly, it will provide an overview of study characteristics, clinical profiles, care delivery, outcomes, and screening tools embedded in the care model. Thirdly, this review will help inform the existing approach and effort to develop new model(s) of care for frail older people in the ED. Furthermore, the results will guide future research towards developing, implementing, and evaluating appropriate model of care tailored to the healthcare system in the emergency context. Despite the rigor applied to this scoping review protocol, potential limitations may eventuate due to resourcing considerations and the nature of the scoping review. First, there may be studies from other countries published in languages other than English which will not be captured. Second, there may be other evidence published in local databases in different countries and grey literature (e.g., government reports) which will not be captured. The protocol amendment will be documented with version control if there are any changes during the process of the review. The results of this review will be presented at international, national, and local health conferences and submitted for publication in a peer-reviewed journal.

## Supplementary Information

**Additional file 1.** PRISMA-P 2015 Checklist.

**Additional file 2.** A Draft Strategy for CINAHL via EBSCOhost and Ovid MEDLINE.

**Additional file 3.** Proposed Data Extraction Form.

## Data Availability

Not applicable

## Abbreviations

ED: Emergency department
GEDI: Geriatric Emergency Department Intervention
GEDI WISE: Geriatric Emergency Department Innovations in Care through Workforce, Informatics and Structural Enhancements models
CARE-PACT: Comprehensive Aged Residents Emergency and Partners in Assessment, Care and Treatment
JBI: Joanna Briggs Institute
PCC: Population, Concept and Context
PRISMA-P check list: Preferred Reporting Items for Systematic Reviews and Meta-analysis Protocols
PRISMA flow diagram: The Preferred Reporting Items for Systematic Reviews and Meta-analysis
NHMRC: National Health and Medical Research Council
