# Supplementary material for: Models of care for frail older persons who present to the emergency department: a scoping review protocol
Source: Syst Rev. 2020 Dec 5;9:280. doi: 10.1186/s13643-020-01534-z (PMC7719249; doi:10.1186/s13643-020-01534-z)
Supplement: Supplementary file 2 — Additional file 2. A Draft Strategy for CINAHL via EBSCOhost and Ovid MEDLINE. [file 13643_2020_1534_MOESM2_ESM.docx]

**Additional file 2. A Draft Strategy for CINAHL via EBSCOhost and Ovid MEDLINE**

| **Search ID#** | **Search Terms (CINAHL)** |
| --- | --- |
| S1 | (MH "Emergency Medical Services") OR (MH "Emergency Service") |
| S2 | TI ("accident and emergency" or "A&E" or "ED" or "A and E") OR AB ("accident and emergency" or "A&E" or "ED" or "A and E") |
| S3 | TI ((emergency N2 (unit or units or room or rooms or ward or wards or department* or service*)) OR AB ((emergency N2 (unit or units or room or rooms or ward or wards or department* or service*)) |
| S4 | (MH "Frail Elderly") OR (MH "Geriatric Functional Assessment") OR (MH "Aged, 80 and Over") OR (MH "Aged") OR (MH "Aged, Hospitalized") |
| S5 | TI (frail or geriatric* or elderly) OR AB (frail or geriatric* or elderly) |
| S6 | TI ((older N2 (patient* or adult* or people or person*)) OR AB ((older N2 (patient* or adult* or people or person*)) |
| S7 | (MH "Geriatric Assessment") |
| S8 | (MH "Health Care Delivery, Integrated") OR (MH "Health Care Delivery") |
| S9 | TI ((model* N2 (service* or care)) OR AB ((model* N2 (service* or care)) |
| S10 | TI ((service* N2 (framework* or infrastructure)) OR AB ((service* N2 (framework* or infrastructure)) |
| S11 | TI ((service* N2 (initiativ* or configurat* or deliver* or capabilit*)) OR AB ((service* N2 (initiativ* or configurat* or deliver* or capabilit*)) |
| S12 | TI ((intervention* N2 (target* or service* or strateg*)) OR AB ((intervention* N2 (target* or service* or strateg*)) |
| S13 | S1 OR S2 OR S3 |
| S14 | S4 OR S5 OR S6 OR S7 |
| S15 | S8 OR S9 OR S10 OR S11 OR S12 |
| S16 | S13 AND S14 AND S15 |
| S17 | S13 AND S14 AND S15  Limiters - Published Date: 2009-onwards; English Language |

| **#** | **Searches (OVID MEDLINE)** |
| --- | --- |
| 1 | Emergency Medical Services/ |
| 2 | Emergency Service, Hospital/ |
| 3 | ("accident and emergency" or "A&E" or "ED" or "A and E").tw. |
| 4 | (emergency adj2 (unit or units or room or rooms or ward or wards or department* or service*)).tw. |
| 5 | Aged/ or Geriatrics/ or Frail Elderly/ |
| 6 | "Aged, 80 and over"/ |
| 7 | (frail or geriatric* or elderly).tw. |
| 8 | (older adj2 (patient* or adult* or people or person*)).tw. |
| 9 | "delivery of health care"/ or "delivery of health care, integrated"/ |
| 10 | Models, Organizational/ |
| 11 | (model* adj2 (service* or care)).tw. |
| 12 | (service* adj2 (framework* or infrastructure)).tw. |
| 13 | (service* adj2 (initiativ* or configurat* or deliver* or capabilit*)).tw. |
| 14 | (intervention* adj2 (target* or service* or strateg*)).tw. |
| 15 | 1 or 2 or 3 or 4 |
| 16 | 5 or 6 or 7 or 8 |
| 17 | 9 or 10 or 11 or 12 or 13 or 14 |
| 18 | 15 and 16 and 17 |
| 19 | limit 18 to (english language and yr="2009 - onwards") |
