# Supplementary material for: Models of care for frail older persons who present to the emergency department: a scoping review protocol
Source: Syst Rev. 2020 Dec 5;9:280. doi: 10.1186/s13643-020-01534-z (PMC7719249; doi:10.1186/s13643-020-01534-z)
Supplement: Supplementary file 3 — Additional file 3. Proposed Data Extraction Form. [file 13643_2020_1534_MOESM3_ESM.docx]

**Additional file 3. Proposed Data Extraction Form**

| **Reviewer:** | |
| --- | --- |
| **Date** | |
| **Key elements** | **Reviewer’s response** |
| Author(s), Year, Country  (NHMRC level of evidence) |  |
| Research Design |  |
| Aim/Purpose |  |
| Study population/Sample size/Time frame of study |  |
| Data collection/Recruitment procedure |  |
| Definition of frailty |  |
| Measures/Screening tools used within the model of care for frail older people |  |
| Types of model of care (e.g., nurse-led model and medical doctor-led model) |  |
| Demographic profile (age, gender and place of residence) |  |
| Clinical Profile (model of arrival, reasons for presentation, time of day, triage category and ICD-10-CM diagnosis code) |  |
| Care delivery (referrals, consultations, follow up and diagnostic tests) |  |
| Outcomes (waiting time to be seen by a doctor, ED length of stay, discharged/admitted and mortality) |  |
